# Targeted Transcriptomic Analysis of C57BL/6 and BALB/c Mice During Progressive Chronic *Toxoplasma gondii* Infection Reveals Changes in Host and Parasite Gene Expression Relating to Neuropathology and Resolution

**DOI:** 10.3389/fcimb.2021.645778

**Published:** 2021-03-18

**Authors:** Kristina V. Bergersen, Ashli Barnes, Danielle Worth, Clement David, Emma H. Wilson

**Affiliations:** ^1^ Division of Biomedical Sciences, School of Medicine, University of California, Riverside, Riverside, CA, United States; ^2^ NanoString Technologies, Seattle, WA, United States

**Keywords:** Toxoplasma, neuropathology, chronic infection, neurons, inflammation

## Abstract

*Toxoplasma gondii* is a resilient parasite that infects a multitude of warm-blooded hosts and results in a lifelong chronic infection requiring continuous responses by the host. Chronic infection is characterized by a balanced immune response and neuropathology that are driven by changes in gene expression. Previous research pertaining to these processes has been conducted in various mouse models, and much knowledge of infection-induced gene expression changes has been acquired through the use of high throughput sequencing techniques in different mouse strains and post-mortem human studies. However, lack of infection time course data poses a prominent missing link in the understanding of chronic infection, and there is still much that is unknown regarding changes in genes specifically relating to neuropathology and resulting repair mechanisms as infection progresses throughout the different stages of chronicity. In this paper, we present a targeted approach to gene expression analysis during *T. gondii* infection through the use of NanoString nCounter gene expression assays. Wild type C57BL/6 and BALB/c background mice were infected, and transcriptional changes in the brain were evaluated at 14, 28, and 56 days post infection. Results demonstrate a dramatic shift in both previously demonstrated and novel gene expression relating to neuropathology and resolution in C57BL/6 mice. In addition, comparison between BALB/c and C57BL/6 mice demonstrate initial differences in gene expression that evolve over the course of infection and indicate decreased neuropathology and enhanced repair in BALB/c mice. In conclusion, these studies provide a targeted approach to gene expression analysis in the brain during infection and provide elaboration on previously identified transcriptional changes and also offer insights into further understanding the complexities of chronic *T. gondii* infection.

## Introduction

*Toxoplasma gondii* is a resilient parasite that infects ~ 10% of the population older than 6 years in the United States alone and more than 60% of populations in other countries worldwide (Prevention, 2018). Following a period of systemic infection and inflammation, the parasite is sequestered within neurons in the brain creating a lifelong infection. A requirement for continuous peripheral inflammation including CD4 and CD8 T cells is required to prevent uncontrolled parasite replication and potentially fatal pathology (Gazzinelli et al., 1992). Despite the presence of parasites and inflammation, infection is subclinical. However, infection in the immunocompromised can result in reactivation of the parasite, leading to Toxoplasmic encephalitis.

Previous data have reported broad transcriptomic changes in the brain of *T. gondii*-infected mice at both acute and chronic time points (Tanaka et al., 2013; Schneider et al., 2019; Hu et al., 2020; Li S. et al., 2020). However, these studies often focus on one specific time point in chronic infection, and they fail to explore transcriptomic changes that occur over the course of infection that are important to determine directional change. The parasite itself goes through genetic changes in order to convert from a fast-replicating tachyzoite during acute systemic infection to a cyst-forming bradyzoite during the early stage of chronic infection after crossing the blood brain barrier (Lachenmaier et al., 2011; Feustel et al., 2012; Hong et al., 2017; Radke et al., 2018). At this early chronic stage, CNS-resident cells begin actively recruiting peripheral immune cells in response to the presence of the parasite, which is still transitioning from tachyzoite to bradyzoite to cyst. As infection progresses and reaches the mid-chronic stage, cyst numbers stabilize as the majority of parasites in the brain are now slow-replicating bradyzoites. At this point in chronicity, both innate and adaptive peripheral immune cells have been in the brain for some time and are constantly maintaining the balance between pro- and anti-inflammatory cytokine production. In addition, subtle neuropathology is commonly seen as noted by changes in morphology and function of both infected and non-infected neurons and changes in glia (David et al., 2016; Graham et al., 2020). Control of infection is maintained for the lifetime of the host and pathology at this stage can vary depending on the genetics of the parasite as well as the background of the host (Liesenfeld et al., 1996; Denkers, 1999; Lee and Kasper, 2004; Lilue et al., 2013; Mukhopadhyay et al., 2020). These changes in parasite phenotype, host immune response, and neuropathology have yet to be fully explored in the context of progressive chronic infection.

The lifelong presence of cysts within neurons and continuous neuroinflammation gives ample opportunity for *T. gondii* infection to initiate significant changes in the brain. Indeed, many studies have demonstrated significant alterations in rodent behavior including attraction to cat urine postulated to be parasite manipulation to maximize the chances of return to the definitive host (Berdoy et al., 2000; Vyas et al., 2007; Boillat et al., 2020). Additionally, infected mice also show significant changes in behavior in an elevated plus maze (David et al., 2016). Such changes may be related to direct and indirect modulation of neurotransmitters including imbalances in excitatory and inhibitory signaling and the production of dopamine (Webster and McConkey, 2010; Prandovszky et al., 2011; Brooks et al., 2015; David et al., 2016; Ngô et al., 2017). However, the presence of the parasite is hard to unlink from the degree of neuroinflammation and any changes in host behavior is likely as much to do with cytokine signaling as the presence of the parasite (Boillat et al., 2020).

To address the complex interplay of infection, inflammation and neurochemistry, there have been several studies investigating transcriptional changes related to chronic CNS Toxoplasma infection. RNAseq analysis of *in vitro* cultured neurons, astrocytes, fibroblasts and skeletal muscle cells tested infection-induced changes in cell -specific transcriptomes and indicated a host-cell dependent genetic basis for spontaneous cyst formation in neurons and skeletal muscle cells (Swierzy et al., 2017). A comprehensive transcriptomic and proteomic study of human brain tissue including those congenitally infected suggests *T. gondii* infection alters neurodevelopment, plasticity, and disease association in various neural and immune networks (Ngô et al., 2017). Although not as clinically relevant, murine studies are robust and have recently underlined the dominance of immune changes in the brain following infection with oocysts of the Prugniaud strain revealing activated pathways of metabolism and biosynthesis at both acute and chronic stages of infection (Jia et al., 2013; Hu et al., 2020).

As with all infections, host genetic background plays an important role in disease and despite the ability of Toxoplasma to establish itself in almost all mammalian hosts, it has been known for some time that the development of disease is highly dependent on mouse strain, recently reviewed by Mukhopadhyay (Blackwell et al., 1993; Johnson et al., 2002; Resende et al., 2008; Mukhopadhyay et al., 2020). Indeed, continuous culturing of Toxoplasma strains *in vivo* rely upon resistant and susceptible strains of mice to maintain virulence and cyst formation, something vitally important to maintain life cycle competency (Goerner et al., 2020). Thus, in the common lab strains of mice, C57Bl/6 are susceptible to disease with greater parasite burden and inflammation while BALB/c mice are resistant to encephalitis dependent on MHC presentation of the antigen GRA6 (Jia et al., 2013; Tanaka et al., 2013; Ngô et al., 2017; Hu et al., 2020; Li S. et al., 2020). These striking differences may provide an opportunity to model disease processes in the brain however little direct comparisons of the kinetics of this host response have been conducted.

In this study, we utilize a targeted approach to analysis gene expression and investigate >1500 genes associated with neurological and immunological processes by direct counting of RNA transcripts without the need for amplification *via* NanoString technology (Geiss et al., 2008). We determine the changes in these host genes over time in parallel with changes in *T. gondii* developmental-specific genes. In addition, we compare side-by-side gene expression changes between susceptible (C57BL/6) and resistant (BALB/c) mouse strains to address possible pathological versus repair signatures of neuroinflammation. Results demonstrate a dramatic shift in both previously demonstrated and novel gene expression relating to neuropathology, inflammation, and neuroinflammation as chronic infection progresses and reveals possible pathways of inflammation resolution.

## Materials and Methods

### Animals and Infections

*Animals.* All research was conducted in accordance with the Animal Welfare Act, and all efforts were made to minimize suffering. All protocols were approved by the Institutional Animal Care and Use Committee (IACUC) of the University of California, Riverside. Female WT C57BL/6 and BALB/c mice were obtained from Jackson Laboratories and were maintained in a pathogen-free environment in accordance with IACUC protocols at the University of California Riverside.

*Infections.* The ME49 strain of *T. gondii* was maintained in cyst form by continuous passaging in SW and CBA background mice. Female 6-8 week old WT C57BL/6 and WT BALB/c mice were infected with 10 ME49 cysts per mouse in 200μl of sterile 1x PBS solution *via* intraperitoneal (IP) injection. Naïve controls received 200µl of sterile 1x PBS solution *via* intraperitoneal (IP) injection. This infection protocol regularly leads to ~3000 cysts in the brains of infected mice by 4 weeks post infection (Goerner et al., 2020).

### DNA Extraction and Parasite Burden Quantification

Parasite burden in brain was quantified as previously described (Goerner et al., 2020). Briefly, DNA from naïve and 2, 4, and 8-week infected half brains of C57BL/6 (n=4/group) and BALB/c (n=5/group) mice was extracted and purified using a High Pure PCR Template Prep Kit (Roche). DNA concentration of each sample was determined via NanoDrop, and all DNA was normalized to 12.5ng/µl before amplification. Parasite burden was measured by amplifying the B1 gene of *T. gondii* by RT PCR.

### RNA Extraction

RNA from brain lysate of naïve, 2 week infected, 4 week infected, and 8 week infected C57BL/6 and BALB/c mice (n=3 for all groups) was collected using Trizol extraction method. At each time point, mice were sacrificed and perfused intracardially with 20mL of sterile 1x PBS. Whole brain was dissected and homogenized in 1mL Trizol. Phase separation was completed by adding 200µl of chloroform and incubating at room temperature for 2-3 minutes. Samples were centrifuged at 12,000xg for 15 minutes at 4°C, and upper aqueous phase was collected followed by RNA precipitation using 500µl isopropyl alcohol. Samples were incubated at room temperature for 10 minutes followed by centrifugation. RNA pellet was washed with 1mL of 75% ethanol twice. All supernatant was discarded and RNA pellet was dissolved in 100µl of nuclease free water. RNA concentration for each sample was determined using a NanoDrop 2000, and RNA samples normalized to a concentration of 10ng/μL in molecular grade water.

### NanoString nCounter Gene Expression Assays and Analysis

*Gene Expression Assays.* nCounter gene expression assays (NanoString Technologies) were performed for each of the following NanoString panels: Neuropathology, Neuroinflammation, and Inflammation+CustomizedPLUS covering a total of 1530 targeted genes including 10 parasite-specific genes: *ROP18, GDA1/CD39, ADF, GRA12, SRS22A, TUBA1, SRS35A, SRS44, BAG1*, and *LDH2* (Geiss et al., 2008). *T. gondii* genes included in custom PLUS codeset were selected based on the percent expression in tachyzoite and bradyzoite growth stages (90% expression in one stage and 0% in other stage) using previous studies differentiating growth stages and ToxoDB (Goerner et al., 2020). Briefly, panel codeset probes were hybridized with 150ng of total RNA per brain over 18hr at 65C according to NanoString protocol. Inclusion of a customized PLUS codeset in the Inflammation panel required additional Reporter and Capture Plus codesets to be added during the hybridization reaction (NanoString User Manual C0019-08). Hybridized RNA was then diluted in molecular grade water and loaded into nCounter SPRINT cartridge (NanoString), placed into nCounter SPRINT Profiler, and quantified. RNA-conjugated probes were counted *via* NanoString Sprint Profiler technology.

#### nSolver and Advanced Analysis

Results from each panel were merged into one data file for comparative analysis, normalized in nSolver following best practices, and analyzed using nSolver and Advanced Analysis software according to previously published protocols (Danaher et al., 2017). nSolver-generated heat maps were created using normalized (merged) data and agglomerative clustering, a bottom-up form of hierarchical clustering (NanoString User Manual C0019-08). For Advanced Analysis, normalized merged data was used (NanoString User Manual 10030-03). Differential expression (DE) analysis was performed to identify specific targets that exhibit significantly increased or decreased expression in response to naïve control values or, in the case of BALB/c C57BL/6 comparison, to C57BL/6 control values at each time point. Gene set analysis was run to determine the change in direction of regulation within each pre-defined gene set relative to naïve controls. Global significance scores, a summary T-statistic used to measure change (NanoString User Manual 10030-03) were calculated, and the directed global significance scores were expressed *via* heatmap. Cell type profiling module analysis was conducted to determine the relative abundance in classically activated cell types during infection using genes assigned to each cell type: T cells (*CD3e, CD6, CD3g, TRAT1*, and *CD3d*), CD8+ T cells (*CD8a*), macrophages (*CD68* and *CD84*), and microglia (*GPR84, LRRC25, IRF8, NCF1, TNF, TLR2*, and *TNF.1*). NanoString cell type profiling is a validated method to measure relative abundance of up to 24 different immune cell types, with high concordance to flow cytometry (Danaher et al., 2017). Pathway analysis was also conducted to determine overall changes in pathways based on the first principal component of the targets within a pathway as annotated by NanoString (NanoString User Manual 10030-03). Direction of pathway change (up- or downregulated) was determined by cross referencing the pathway score with the corresponding volcano plot for that pathway. Summary pathway score plot colors are based on calculated scores and are represented as downregulation (blue) to upregulation (orange). Statistical significance was determined using R software. All data are available on Gene Expression Omnibus (GEO).

#### Separate Statistical Analyses

Normalized Log_2_ scores for *T. gondii*-specific genes were used in fold changes analysis of tachyzoite-associated genes and bradyzoite-associated genes compared to naïve controls in Prism. In addition, results from cell type profiling modules were placed into Prism and evaluated at each time point. Violin plots were created in a similar fashion by analyzing normalized Log_2_ scores of specific probes to show the rounded distribution of data. Statistical significance was determined by 2-tailed, unpaired Student’s *t*-test and One-Way ANOVA with multiple comparisons (p-value < 0.05).

## Results

### Chronic *Toxoplasma gondii* Infection Stimulates a Dramatic Shift in Gene Expression as Infection Progresses From Early to Late Chronic Stages

**Figure 1:**
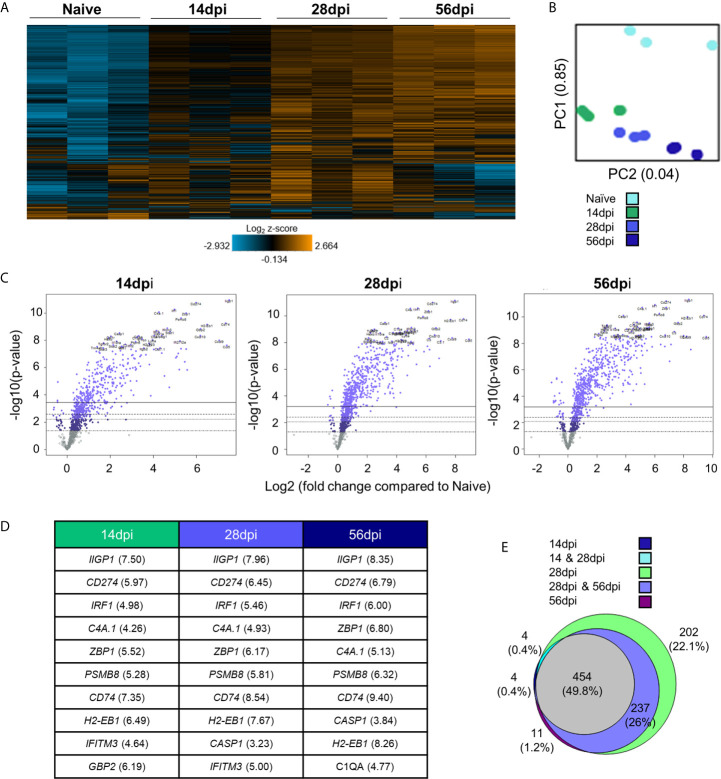
Chronic *T. gondii* infection stimulates a dramatic shift in gene expression as infection progresses from early to late chronic stages. **(A)** Heat map of all genes from merged Neuropathology, Neuroinflammation, and InflammationPLUSCustomCodeset panels. Gene expression depicted from low expression (blue) to high expression (orange). Heat map generated from normalized gene expression data using nSolver software. **(B)** PCA plot of biological replicates at all time points. Numbers on axes represent percentage of variation in that component. **(C)** Differential expression analysis results of 14 dpi, 28 dpi, and 56 dpi compared to Naïve control values demonstrates the majority of genes as upregulated. Top 25 genes most significantly upregulated identified based on fold change (x-axis) vs. p-value (y-axis). Four adjusted p-value cutoffs in each plot are as follows from bottom (dashed line) to top (solid line): <0.50, <0.10, <0.05, <0.01. **(D)** Table of 10 most significantly upregulated genes at each time point (from C.) Numbers in parentheses represent fold-change of gene. **(E)** Overlap between differentially expressed genes plotted in **(C)**. Numbers and percentages in diagram based on genes significantly upregulated with adjusted p-value cutoff <0.05.

To determine broad transcriptomic changes that occur over the course of infection, RNA from infected C57BL/6 mouse brains was harvested at day 14, 28 and 56 days post infection (dpi) representing time points associated with transitionary, stable and late chronic infection respectively. RNA was hybridized with NanoString panel-specific codesets using two panels of genes specific for inflammation and neuropathology, and RNA-conjugated probes were counted *via* NanoString Sprint Profiler technology. Resulting gene expression changes compared to calculated control z-scores are demonstrated in **Figure 1**. Merged data analysis of NanoString neuropathology, neuroinflammation, inflammation, and a Toxoplasma-specific custom codeset probes using basic nSolver software demonstrate dramatic shifts in gene expression (**Figure 1A**). The majority of genes relating to neuropathology, neuroinflammation, and inflammation switch completely from low expression (blue) compared to control z-scores in an uninfected state to high expression (orange) by late chronic infection, signifying an 8-10 fold change in expression. This significant switch is seen almost in entirety by the mid-chronic stage of infection when cysts are prevalent in the brain. Contrastingly, a multitude of gene expression changes are still occurring at the early chronic stage correlating with tachyzoite entry and active cyst formation (Dupont et al., 2012; Cabral et al., 2016; Mendez and Koshy, 2017). Biological replicates in each time point generally exhibit close clustering with 14dpi showing the greatest variation in line with a transitioning state between acute and chronic infection (**Figure 1B**).

Consistent with a powerful infection in an otherwise immune privileged site, differential expression analysis reveals that at all timepoints, the majority of genes significantly altered by infection are upregulated as compared to naïve controls (**Figures 1C–E**; **Supplementary Table 1**). At 14dpi, the majority of genes that experience changes in expression undergo significant upregulation despite CNS infection not being fully established. At this early stage, many genes exhibit fold changes up to 7-fold (*CCL5, CD74*) and this only increases at later chronic timepoints, reaching up to 8-fold change at 28dpi and up to 10-fold change at 56dpi.

Comparison of genes that are significantly altered as determined by differential expression analysis reveals both conserved gene expression changes across all timepoints as well as time point-specific changes (**Figure 1E**). At 28dpi (light green), there are 202 genes that have significant changes in expression (22% of total) that are specific to this mid-chronic stage of infection. This number is significantly greater than the stage-specific changes seen at 14dpi (dark blue; 4 genes = 0.4%) and 56dpi (dark purple; 11 genes = 1.2%). There are no shared differentially expressed genes (DEGs) between the early (14dpi) and late (56dpi) chronic stages and very few shared genes (light blue, 4 genes = 0.4%) between the early (14dpi) and mid- (28dpi) chronic stages of infection. Consistent with establishment of infection, mid- (28dpi) and late (56dpi) chronic stages share a far greater number of DEGs (light purple, 237 genes = 26% of total), signifying a similar infection phenotype between later stages of infection as chronicity is firmly established and maintained. Of all genes expressed above background levels included in the merged analysis, 454 genes (grey, 49.8% of total) are significantly altered at all timepoints. 

### *Toxoplasma gondii* Stage-Specific Gene Expression Correlates With Host Gene Changes

**Figure 2:**
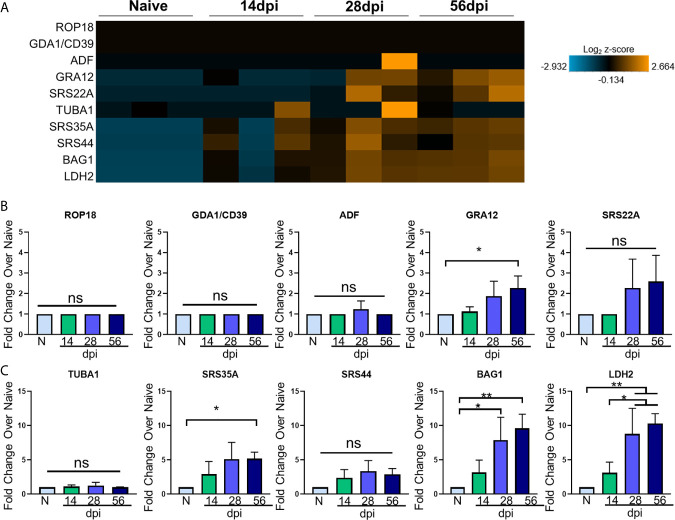
T. gondii stage-specific gene expression correlates with host gene changes. **(A)** Heat map of *T. gondii*-specific genes from merged Neuropathology, Neuroinflammation, and InflammationPLUSCustomCodeset panels. Gene expression depicted from low expression (blue) to high expression (orange). Heat map generated from normalized gene expression data using nSolver software and Background Thresholding to account for lower parasite gene counts. **(B, C)** Fold change compared with naive of tachyzoite genes **(B)** and bradyzoite genes **(C)** from normalized gene counts. Significance determined by One-Way ANOVA using Multiple Comparisons (*p-value <0.05, **p-value <0.01.). ns, not significant.

Toxoplasma goes through several phases of development and in the intermediate mammalian host, two stages dominate: i) a fast-replicating tachyzoite responsible for dissemination, cell lysis, and acute pathologies and ii) the bradyzoite that replicates slowly, and forms cysts in neurons (Montoya and Liesenfeld, 2004). How these classical Toxoplasma signatures change in relation to the host transcriptome over the course of *in vivo* infection has not been explored in depth. The broad significant changes in host genes following infection may represent a triggering of inflammation cascades and neurological remodeling that is independent of infection, or they could be closely tied to the development of the parasite niche in the brain. To determine the relationship between host and parasite genetic changes, Toxoplasma stage-specific genes were selected based on their stage specificity and frequency of expression in RNAseq data sets of Me49 bradyzoites, tachyzoites, and late-stage bradyzoites as seen in ToxoDB and in recent publications (Goerner et al., 2020). These genes were incorporated into the NanoString analysis *via* the use of custom codesets, and changes in expression of these genes were analyzed at each time point. To further elucidate *T. gondii* stage-specific gene expression changes across the course of infection, fold changes of tachyzoite-associated genes and bradyzoite-associated genes compared to naïve controls were analyzed *via* OneWay ANOVA for significance. Normalized parasite gene counts for each sample at each time point were used to determine fold change. To verify normal progression of infection, parasite B1 amplification was conducted (**Supplementary Figure 1**). Visualization of parasite-specific gene expression changes correlates with development of chronic and sustained infection (**Figures 2A–C**; **Supplementary Figure 1**).

Most tachyzoite associated genes (top 5 genes in heat map) are expressed at or around background levels in the brain and remain so for the duration of infection (**Figure 2A**). *ROP18, GDA1/CD39*, and *ADF* are constitutively expressed across all developmental stages according to ToxoDB but are never detectable above background here and may indicate limitations in sensitivity compared to the abundance of host genetic material (**Figures 2A, B**). These are all genes constitutively expressed in varying stages of Me49 strain development, and their expression is not altered at any point during chronic infection (**Figure 2B**). Therefore, although C57BL/6 mice are susceptible to infection exhibiting increasing parasite burden over time, the stable expression of *ROP18, GDA1/CD39*, and *ADF* in this study suggest that even at 56dpi, there are not classical indicators of parasite reactivation and bradyzoite genes continue to dominate.

The genes *GRA12* (Krishnamurthy and Saeij, 2018) and *SRS22A* (Michelin et al., 2009) help with formation of the parasitophorous vacuole (PV) membrane that protects the parasite during both acute and chronic infection, and *GRA12* is one of the genes that orchestrates cyst formation in the transition from tachyzoite to bradyzoite and is seen in the 99^th^ percentile in both tachyzoites and bradyzoites (Guevara et al., 2019). Our data show *GRA12* increasing by 2.5-fold at 56 days post infection, signifying the presence of non-replicating parasites in the brain while *SRS22A* remains unchanged compared to naïve controls (**Figure 2B**).

In addition to tachyzoite associated genes, early- and late-stage bradyzoite genes were selected for this analysis based on ToxoDB expression values. *SRS35A*, also known as *SAG4*, is a surface protein expressed above the 95^th^ percentile in both early and late-stage bradyzoites (Zhou and Wang, 2017). *SRS44*, also known as *CST1*, is a cyst wall component that is expressed above the 89^th^ percentile in both bradyzoite stages and maintains the integrity of the cyst during chronic infection (Tomita et al., 2013). The bradyzoite-specific antigen *BAG1*, also thought to aid in cyst formation (Zhang Y.W. et al., 1999), is expressed at the 91^st^ and 100^th^ percentile in early and late stage bradyzoites respectively. *LDH2*, one of the main controllers of bradyzoite differentiation (Abdelbaset et al., 2017) is expressed between the early and late bradyzoite stages of growth.

All of these genes increase in percentile of expression in early- and/or late-stage bradyzoite stages of growth compared to tachyzoites. In contrast to the lack of tachyzoite genes, kinetics of these selected bradyzoite genes follow a pattern of increasing expression from 14dpi to 56dpi (**Figures 2A, C**). Fold change analysis of bradyzoite associated genes reveal significant increases of the bradyzoite specific *SRS35A, BAG1* and *LDH2* as infection progresses (**Figures 2A, C**). *SRS35A* shows a 5-fold change compared to naïve controls at 56 days post infection*. SRS44* experiences an increasing trend but does not reach significance. *BAG1* demonstrates between a 5-10-fold increase in expression at 28 and 56 days post infection, supporting the presence of bradyzoites in the brain at these later chronic stages. *LDH2* shows an approximately 10-fold increase at 28 and 56 days post infection. Expression of *LDH2* at the mid- and late-chronic stage is also significantly higher compared to expression at 14dpi.

Thus, several ToxoDB-identified constitutively expressed tachyzoite genes were unchanged between the early and late chronic stages of infection based on their expression at or below background levels. However, by 28dpi a significant pattern of Toxoplasma gene expression has been established dominated by late-stage bradyzoite specific genes (Hong et al., 2017; Garfoot et al., 2019; Goerner et al., 2020). Interestingly, this pattern remains mostly unchanged 4 weeks later at 56dpi with the exception of *GRA12* that may support renewed cyst formation at this later stage (Watts et al., 2015). Taken together, the kinetics of parasite-specific gene expression in the C57BL/6 mouse indicate an established chronic infection, minimal tachyzoite replication and a predominantly late-stage bradyzoite phenotype.

### Kinetics of Chronic Infection Demonstrates Classical Immune Cell Activation and Increases in IFNγ Signaling-Related Genes

**Figure 3:**
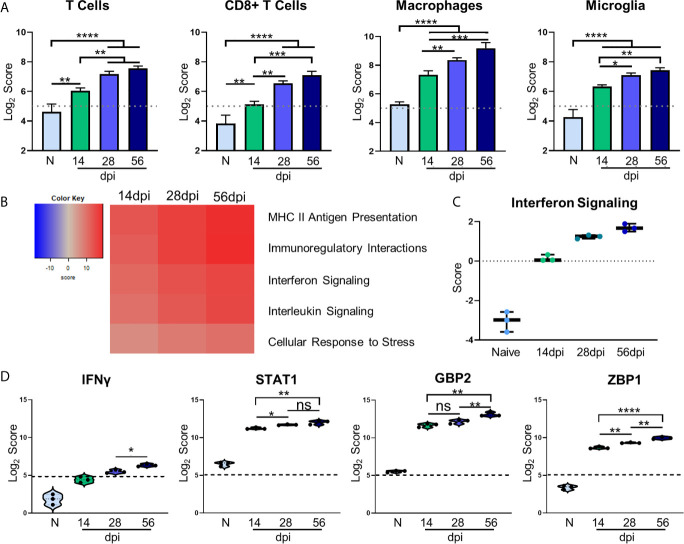
Kinetics of chronic infection demonstrates classical immune cell activation and increases in IFNγ signaling-related genes. Cell-type profiling, global significance analysis, pathway scoring, and individual gene results of merged Neuropathology, Neuroinflammation, and InflammationPLUSCustomCodeset panels. **(A)** log_2_ score plots of infiltrating immune cell types T cells, CD8+ T cells, macrophages, and resident microglia compared to naïve time point control (*p-value < 0.05, **p-value < 0.01, ***p-value < 0.001, ****p-value < 0.0001). All cell types checked for expression above background level of 5 (shown by dashed line). **(B)** Heatmap of directed global significance scores compared to naïve controls based on direction of gene set pathway change. Red denotes gene sets whose genes exhibit extensive over-expression with the covariate, blue denotes gene sets with extensive under-expression. **(C)** Pathway analysis results of Interferon Signaling. Pathway checked for expression compared to background score of 0 (shown by dashed line). **(D)** log_2_ score plots of specific genes relating to IFNγ signaling, specifically *IFNγ*, *STAT1*, *GBP2*, and *ZBP1* compared to naïve time point control (*p-value < 0.05, **p-value < 0.01, ***p-value < 0.001, ****p-value < 0.0001). All genes checked for expression above background level of 5 (shown by dashed line). ns, not significant.

Regulation of inflammation is especially important in the CNS during chronic infection, and a balanced host immune response is vital for survival. The role of T cells in protection against Toxoplasmic encephalitis has been known for some time however, the accumulation of innate immune cells, resident CNS cells and subsets of all of these are still not fully documented (Landrith et al., 2015; Khan et al., 2019). In contrast to RNA-seq, the direct counting of transcripts (Geiss et al., 2008) allows cell type profiling analysis of immune cells based on the counts of particular cell-specific transcripts (Danaher et al., 2017). Results of cell type profiling demonstrate significant changes in abundance of canonical cell types involved in the *T. gondii* immune response (**Figure 3A**). As would be expected all T cells and specifically CD8+ T cells significantly increase in abundance over naive and 14dpi as infection progresses (Hu et al., 2020) (**Figure 3A**). In addition, macrophages and resident microglia also increase in abundance and activation. The increase in these innate immune cell types follows the same pattern as T cells, with macrophages demonstrating the largest increase over time. Both macrophages and microglia exhibit significant increases over naïve at all time points and level off after 28dpi.

To examine the overall directional change of specific infection-associated pathways, gene set analysis (GSA) was performed and directed global significance scores were analyzed. As seen in **Figure 3B**, classical inflammatory response pathways follow a trend of steady upregulation across all time points supporting the need for a maintained robust immune response. Our pathway analysis results show an anticipated initial increase in IFN signaling during the early chronic stage of infection that consistently increases over time (**Figure 3C**). Downstream IFNγ-dependent mechanisms such as *STAT1* signaling and parasite-killing genes *GBP1* and *ZBP2* are vital to prevent parasite reactivation in the brain (Virreira Winter et al., 2011; Kravets et al., 2012; Hidano et al., 2016; Pittman et al., 2016). To address the kinetics of *IFNγ, STAT1, GBP2*, and *ZBP2* Log_2_ scores of transcript numbers were plotted for each time point (**Figure 3D**). *IFNγ* expression serves as a reassuring control of previously known infection-induced brain changes, results demonstrate low *IFNγ* at 14dpi consistent with lower numbers of T cells at this early stage in the brain (**Figure 3D**, left). This increases as chronicity progresses and reaches peak significance at 56dpi compared to the mid-chronic stage. Despite this conservative *IFNγ* expression, it is highly effective with large increases in IFNγ-dependent signaling including *STAT1*. Corresponding to increased IFNγ-dependent signaling, *STAT1* expression is increased 2-fold in the brain at 14dpi and continues to increase as infection progresses through the mid-chronic stage, levelling off between 28 and 56dpi (**Figure 3D**, second from left). *GBP2* and *ZBP1* expression are also increased at 14dpi compared to naïve controls, but while *GBP2* remains constant until the late stage of infection, *ZBP2* is significantly increased at each time point (**Figure 3D**, right). The differences in timing of expression of these genes suggests stage-specific, IFNγ-dependent mechanisms of parasite control. Taken together, these results demonstrate classical activation of immune cells and infection-associated pathways that supports previous research as well as previously unknown kinetics of IFNγ-dependent signaling mechanisms.

### Kinetic Analysis Reveals Progressive Neuropathological Changes and Previously Unexplored Attempts at Repair During Chronic *Toxoplasma gondii* Infection

**Figure 4:**
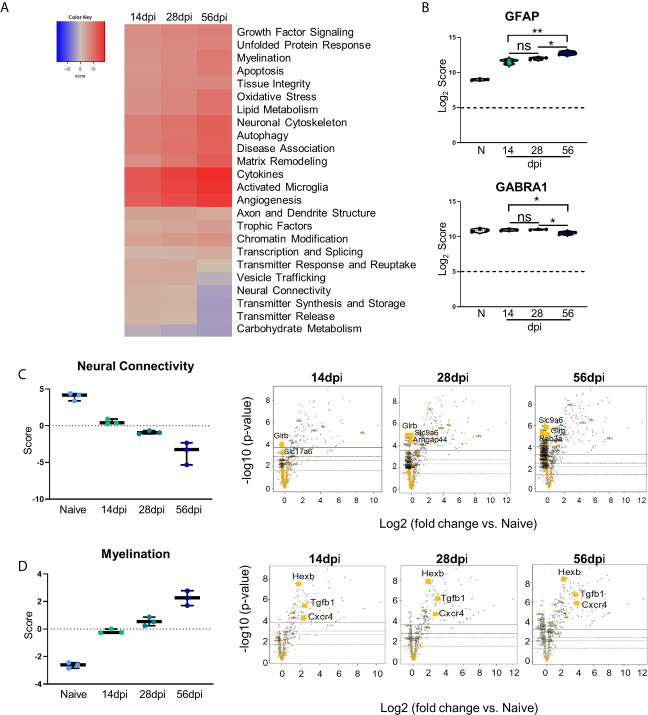
Kinetics of chronic infection reveals progressive neuropathological changes and previously unexplored attempts at repair/maintenance during chronic *T. gondii* infection. Global significance analysis, individual gene, and pathway scoring results of Neuropathology Panel. **(A)** Heatmap of directed global significance scores compared to naïve controls based on direction of gene set pathway change. Red denotes gene sets whose genes exhibit extensive over-expression with the covariate, blue denotes gene sets with extensive under-expression. **(B)** log_2_ score plots of specific genes relating to neuropathology, specifically *GFAP* and *GABRA1* compared to naïve time point control (*p-value < 0.05, **p-value < 0.01). All genes checked for expression above background level of 5 (shown by dashed line). **(C, D)** Pathway analysis and volcano plot results of neural connectivity **(C)** and myelination **(D)** pathways significantly affected during *T. gondii* infection based on analysis. Volcano plot of directed global significance scores for neural connectivity and myelination gene sets. Genes within the selected gene set are highlighted in orange. Top three genes driving directional change identified based on fold change (x-axis) vs. p-value (y-axis). Four adjusted p-value cutoffs in each plot are as follows from bottom (dashed line) to top (solid line): <0.50, <0.10, <0.05, <0.01. ns, not significant.

Changes in neurochemistry in the infected brain have previously been reported including alterations in the excitatory and inhibitory neurotransmitters (Xiao et al., 2013; Brooks et al., 2015; David et al., 2016; Barbosa et al., 2020) that suggest that even in the absence of clinical pathology, there are underlying changes in neuronal structure and connectivity that would be described as pathological. To determine the full extent of genetic changes related to neuropathology, we analyzed a group of genes with known roles in transmitter function, neural connectivity and function, and neural maintenance and repair *via* GSA and pathway analyses. GSA results demonstrate upregulation of most of the Neuropathology panel pathways at each time point while a handful of pathways (namely, Vesicle Trafficking, Neural Connectivity, Transmitter Synthesis and Storage, Transmitter Release, and Carbohydrate Metabolism) experience noticeable downregulation (**Figure 4A**). To confirm worsening of neuropathology *via* the activation of CNS-resident immune cells and decreases in neuronal function, Log_2_ scores of the astrocyte activation gene *GFAP* and the inhibitory neurotransmitter GABA signaling gene *GABRA1* were analyzed (**Figure 4B**). Astrocytic *GFAP* increases in expression initially during the early chronic stage of infection, remains steady between 14 and 28dpi, and then experiences a significant increase in expression by 56dpi (**Figure 4B**, top). Over the course of chronic infection, *GABRA1* remains at levels comparable to naïve controls until 56dpi where it demonstrates a significant decrease (**Figure 4B**, bottom).

As neurons are the primary CNS-resident cell type infected by *T. gondii*, and these cells undergo changes in morphology and function during infection, the pathway of neural connectivity was examined more thoroughly *via* pathway analysis and corresponding volcano plots. Pathway analysis supports GSA results and shows a steady decrease in neural connectivity across all timepoints (**Figure 4C**). Volcano plots of the genes driving the neural connectivity score show increases in significantly downregulated genes as infection progresses corresponding with the lower GSA score at each time point (**Figure 4C**). One gene that drives this downregulation at all time points is *Glrb*, a receptor that functions as a neurotransmitter-gated ion channel (Handford et al., 1996; Ridderbusch et al., 2019). *Slc9a6*, a gene that encodes the protein NHE6 which plays a role in dendritic spine growth (Park et al., 2006; Gao et al., 2019), becomes highly downregulated at the later time points (**Figure 4C**). *Slc17a6* which aids in glutamate uptake (Takamori et al., 2001; Serrano-Saiz et al., 2020) drives downregulation of neural connectivity at 14dpi (**Figure 4C**). In addition to supporting previous findings and in contrast to immune scoring, this data suggests a continuous progression of neuropathology specifically in the areas of neurotransmitter production and function (**Supplementary Table 1**).

GSA and pathway analyses also reveal an increase in pathways associated with myelination (**Figures 4A, D**). The myelination pathway experiences consistent upregulation as infection progresses through chronicity (**Figures 4A, D**). It is well known that myelination of neuronal axons is a critical process not only for neuronal signaling, but also for continued supply of required metabolites to neurons for their survival and function and the process of re-myelination has been seen in various instances of CNS injury and disease (Saab and Nave, 2017; Wang F. et al., 2018). Throughout all stages of infection, the increase in the myelination pathway is driven by the genes *Hexb*, *Tgfb1*, and *Cxcr4* (**Figure 4D**). These results demonstrate an increase in genes associated with myelination and support increased attempts at repair of neuropathology.

### Kinetics of *Toxoplasma gondii* Infection Reveals Significant Alteration of Novel Genes

**Figure 5:**
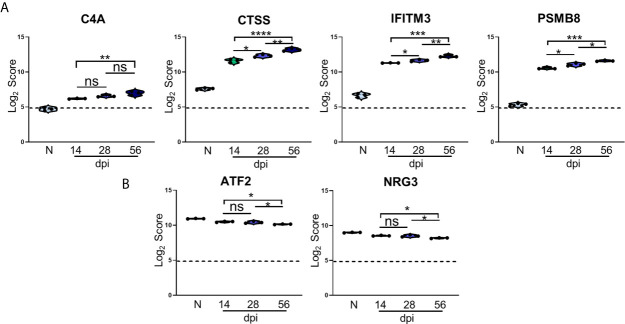
Kinetics of *T. gondii* infection reveals significant alteration of novel genes. **(A, B)** log_2_ score plots of specific novel genes from merged Neuropathology, Neuroinflammation, and InflammationPLUSCustomCodeset panels compared to naïve time point control. **(A)** Violin plot results of most highly upregulated non-canonical genes *C4A*, *CTSS*, *IFITM3*, and *PSMB8* as determined by differential expression analysis. **(B)** Results of most highly downregulated non-canonical genes *ATF2* and *NRG3* as determined by differential expression analysis. All genes shown are expressed above background threshold of 5 (shown by dashed line) and are significantly altered during infection based on differential expression analysis statistics (p-value<0.01). All graphs shown as fold change over background. Significance between timepoints determined by One-Way ANOVA using Multiple Comparisons (*p-value <0.05, **p-value <0.01, ***p-value < 0.001, ****p-value < 0.0001). ns, not significant.

In addition to changes in expression of canonical *T. gondii* infection-associated genes, differential expression analysis also identified significant changes in several genes previously not associated with this parasitic infection. Of the 25 most significantly upregulated genes from all time points compared to Naïve controls, 4 of these genes were determined to be novel in the context of *T. gondii* infection, and their Log_2_ scores were analyzed further (**Figure 5A**).

*C4A* gene expression increases compared to naïve controls beginning at the early stage of chronic infection, and while this expression does not change significantly between 14dpi and 28dpi or 28dpi and 56dpi, expression increases by approximately 1.5 fold overall between the early and late chronic stages (**Figure 5A**). This gene is primarily known for activation of the complement pathway along with *C3A* and *C5A* (Liesmaa et al., 2018; Melbourne et al., 2018; Prasad et al., 2018; Ji et al., 2019). *CTSS* is a member of the peptidase C1 family that encodes for cathepsin S which is expressed by neurons in the CNS (Ji et al., 2018). Similar to *C4A*, *CTSS* expression experiences >1.5 fold change over the course of infection, but this gene is also significantly increased at each chronic time point (**Figure 5A**). *IFITM3*, commonly known for its anti-viral functions, experiences a similar pattern of upregulation as *CTSS*, and over the course of chronic infection is upregulated >1.5 fold overall (**Figure 5A**). *PSMB8* expression increases 2-fold overall and follows the trend of a step-wise increase between each time point (**Figure 5A**). This gene has the greatest fold change in expression compared to naïve controls.

While the majority of novel differentially expressed genes are upregulated over the course of infection, a handful of previously unreported genes are significantly downregulated compared to naïve controls (**Figure 5B**). One such gene is *ATF2*, activating transcription factor 2, which is required to regulate the transcription of the pro-inflammatory cytokine TNFα (Falvo et al., 2000; Tsytsykova and Goldfeld, 2002). *ATF2* decreases significantly from 28dpi to 56dpi after an initial decrease at 14dpi (**Figure 5B**). *NRG3* belongs to the NRG gene family and is the second most common form of NRG found in the adult brain (Paterson et al., 2017). *NRG3* experiences a similar decrease in expression as *ATF2* over the course of chronicity (**Figure 5B**). Taken together, the consistent pattern of change in these newly analyzed genes over the course of infection point to additional targets for understanding the complexity of chronic infection.

### BALB/c Mice Exhibit Differences in Timing and Expression of Genes, Immune Cell Recruitment, and Pathway Activation Compared With C57BL/6 Mice Suggesting Decreased Neuropathology and Enhanced Repair

Host genetics influence the outcome of Toxoplasma infection even if they do not prevent chronic infection. Thus, C57BL/6 mice are considered susceptible with high cyst burden that levels off around 30 days post infection (Burke et al., 1994) while BALB/c mice, another commonly used mouse strain, are more resistant (**Supplementary Figure 1**) (Mukhopadhyay et al., 2020). While the increased resistance to infection in BALB/c mice has previously been linked to enhanced immune response specifically *via* MHC gene expression (Brown et al., 1995), less is known regarding how neuropathology signatures differ between resistant BALB/c mice and the more susceptible C57BL/6 model over the course of chronic infection.

**Figure 6:**
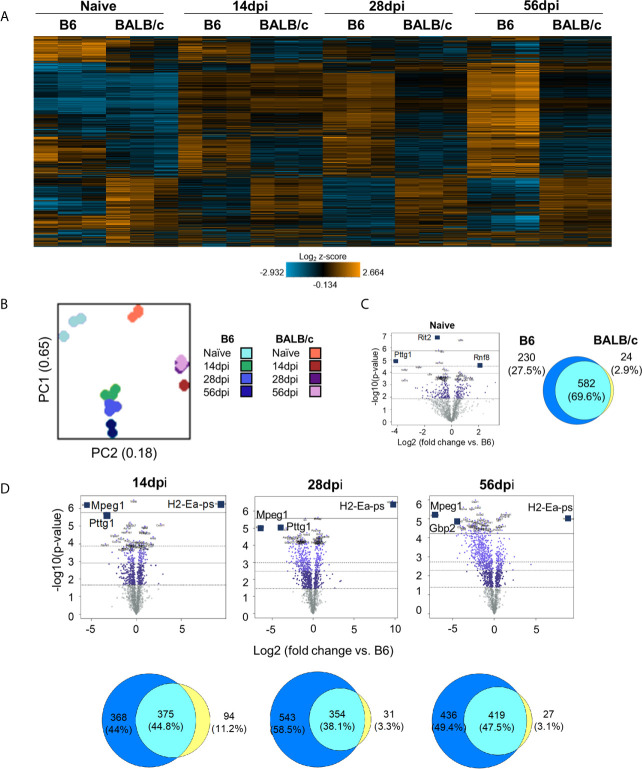
BALB/c mice exhibit differences in timing and expression of genes compared to B6 mice during chronic infection. **(A)** Heat map of all genes from B6vsBALB/c merged Neuropathology, Neuroinflammation, and InflammationPLUSCustomCodeset panels. Gene expression depicted from low expression (blue) to high expression (orange). Heat map generated from normalized gene expression data using Basic nSolver software. **(B)** PCA plot of B6 and BALB/c biological replicates at all time points. Numbers on axes represent percentage of variation in that component. **(C, D)** BALB/c differential expression analysis results and overlap between differentially expressed genes of Naïve **(C)** and infection timepoints **(D)** (14dpi, 28dpi, and 56dpi) compared to B6 control values. Top 3 genes identified based on fold change (x-axis) vs. p-value (y-axis). 4 adjusted p-value cutoffs in each plot are as follows from bottom (dashed line) to top (solid line): <0.50, <0.10, <0.05, <0.01. Numbers and percentages in Venn diagrams based on genes significantly upregulated with adjusted p-value cutoff <0.05.

To compare neuropathology and neuroinflammatory signatures in BALB/c and C57BL/6 mice during chronic infection, NanoString analysis was conducted on BALB/c mouse brain RNA using the same time points of infection as before. Results of merged BALB/c vs. C57BL/6 analysis demonstrate initial differences in gene expression between naïve mice (**Figure 6A**) consistent with previous analysis (Sellers et al., 2011; Yuan et al., 2020). In contrast to B6 mice which generally show a continued increase in gene change over the course of infection, BALB/c mice exhibit the largest change at 14dpi which then returns to patterns similar to naïve at chronic infection. (**Figure 6A**). Principal component analysis (PCA) shows little variation between biological replicates and no divergent point between BALB/c and C57Bl/6 instead gene expression in these different hosts at all time points remain separated. Based on PC1 and PC2 clustering of day 28 and day 56 post infection on the BALB/c background are almost identical (**Figure 6B**). When comparing DEGs between naïve C57BL/6 and BALB/c mice, there is an initial difference in gene expression (**Figure 6C**). While most DEGs (582) are conserved between the 2 strains, the majority of BALB/c genes are downregulated compared to naïve C57BL/6 mice, and there are notably less DEGs that are specific to BALB/c mice (24 BALB/c compared to 230 C57BL/6). At each infection time point, there are between 350-420 genes that remain shared between the 2 mouse strains suggesting conserved gene expression changes relating to neuropathology and neuroinflammation across different mouse species (**Figure 6D**). However, despite this shared number of altered genes, there is a much higher number of DEGs in C57BL/6 mice (368, 543, and 436 genes respectively) compared to BALB/c mice (94, 31, and 27 genes) at each time point. It is important to note that BALB/c mice also have lower numbers of DEGs overall at each time point compared to C57BL/6 mice (**Supplementary Table 2**). The underlying genetic difference between these two strains of mice is observed in the expression of MHC II (*H2-Ea-ps*) and dominates the genes that are upregulated in BALB/c mice (**Figure 6D**). It is not apparent in baseline differences as little MHC II expression occurs in a naïve brain. The overall downregulation of genes following infection of BALB/c mice is dominated by *Mpeg1* and *Pttg1* (**Figure 6D**). These genes are primarily responsible for innate immune responses and apoptosis *via* the p53 pathway (Zhang X. et al., 1999; Bernal et al., 2002; Bai et al., 2018; McCormack et al., 2020; Ni et al., 2020).

**Figure 7:**
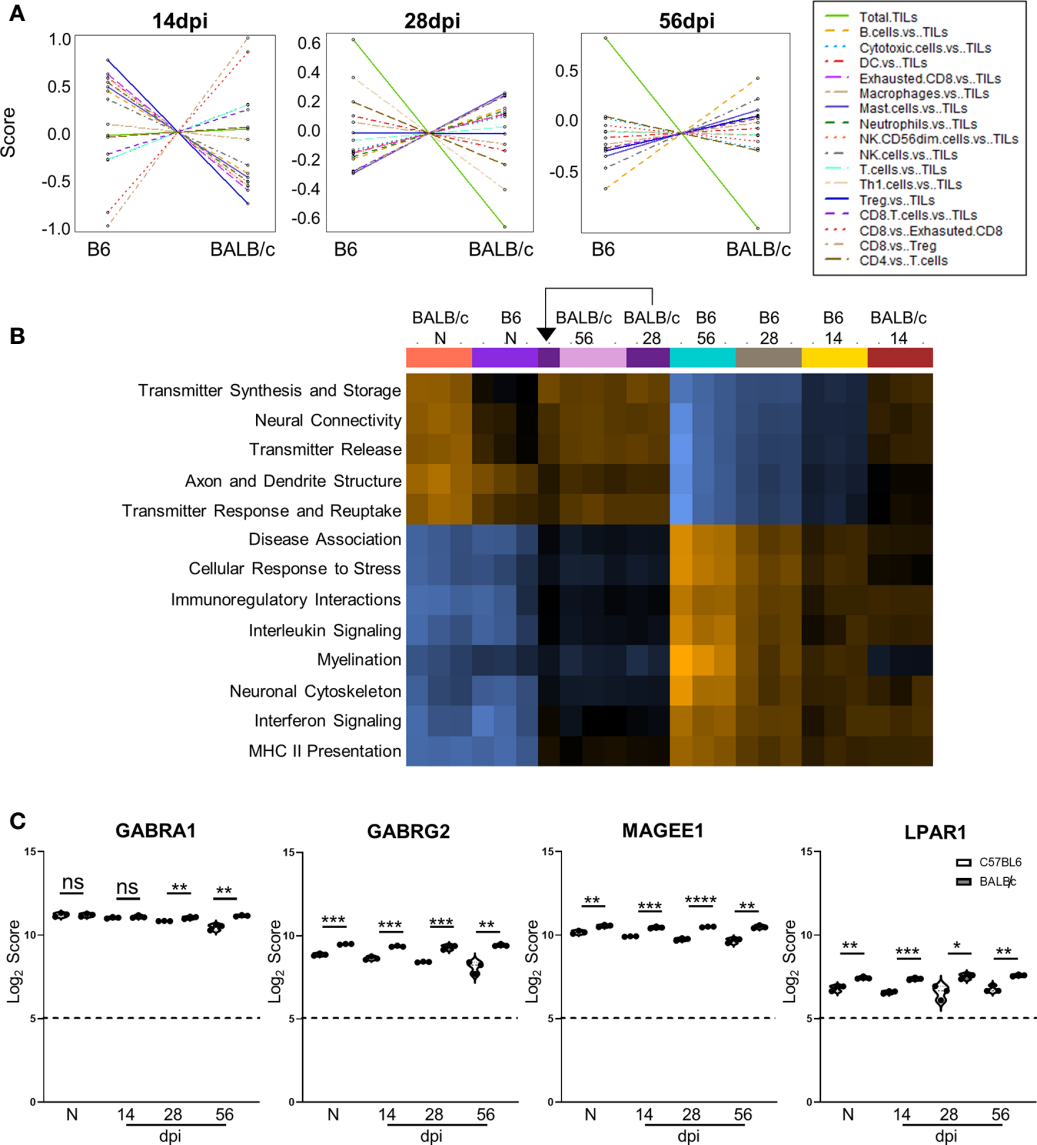
Differences in cell recruitment, pathway activation, and specific regulatory genes demonstrate enhanced control of infection in BALB/c mice. **(A)** Cell type summary plots of recruited cells shown in BALB/c mice vs. B6 mice per time point. **(B)** Pathway score summary plot of BALB/c Vs. B6 mice depicted from downregulation (blue) to upregulation (orange). Summary plot generated by clustering pathways based on similar scores. Samples are arranged in plot according to similarity of pathway score profiles. **(C)** log_2_ score plots of specific genes relating to neuronal health and repair, specifically *GABRA1*, *GABRG2*, *MAGEE1*, and *LPAR1* compared to naïve time point control (* = p-value < 0.05, ** = p-value < 0.01, *** = p-value < 0.001, **** = p-value < 0.0001). All genes checked for expression above background level of 5 (shown by dashed line). Gene selection significance determined *via* differential expression analysis statistics (p-value<0.05). All graphs shown as fold change over background Significance between mouse strains at each time point determined by Unpaired Two-Tailed Student’s t-Test (* = p-value <0.05, ** = p-value <0.01, *** = p-value < 0.001, **** = p-value < 0.0001). ns, not significant..

Based on the differences in differential gene expression between BALB/c mice and C57BL/6 mice, cell type profiling and pathway analyses were conducted to determine effects of cell composition and pathway activation on differences in BALB/c vs. C57BL/6 neuropathology signatures. Cell type profiling demonstrates notable differences in cell composition in the brain across chronic infection between these strains (**Figure 7A**). At each stage, BALB/c mice demonstrate drastically different cell composition overall compared to those of C57BL/6 mice at the same infection stage after comparison to control “Tumor Infiltrating Leukocyte” (TIL) expression as well as other cell types. While not all cell types are consistently up- or downregulated in BALB/c mice at each time point, CD4+ and CD8+ T cells show patterns of decreased and increased amounts respectively (**Figure 7A**) corresponding with previous research demonstrating decreased importance of CD4+ T cells and increased dependence on CD8+ T cell activity in BALB/c chronic infection (Parker et al., 1991; Deckert-Schlüter et al., 1994; Harris et al., 2010). In addition, there are significantly elevated macrophages at 56dpi in BALB/c mice compared to C57BL/6 mice. Notably, total TILs are predominantly downregulated in BALB/c at 28 and 56dpi suggesting less absolute numbers of immune cells and therefore immune cell recruitment overall while TILs are relatively comparable at 14dpi between the mouse strains. This is consistent with increased control of parasite replication in BALB/c (**Supplementary Figure 1**).

To compare pathway score profiles between mouse strains, the heatmap of overall pathway scores was analyzed. This summary plot clusters pathways together based on similar expression patterns and arranges samples based on similarity of pathway expression profiles. When evaluating pathway activation associated with neuropathology, BALB/c mice demonstrate downregulation of neuropathological and inflammatory pathways such as “Disease Association,” “Cellular Stress Response,” “Interleukin Signaling,” “Interferon Signaling,” and “MHC II Presentation” when compared to the same C57BL/6 time points (**Figure 7B**). In addition to decreased neuropathology and inflammatory pathway activation, BALB/c mice also demonstrate upregulation of pathways associated with neurological health and function such as “Transmitter Synthesis and Storage,” “Neural Connectivity,” “Transmitter Release,” “Axon and Dendrite Structure,” and “Transmitter Response and Reuptake” at later chronic time points compared to C57BL/6 mice. While pathway activation patterns are comparable between BALB/c and B6 mice at Naïve and 14dpi, there is a switch in up- and downregulated pathways associated with neuropathology, inflammation, and neurological health at later chronic time points.

To determine if the contrasting pathway activation between BALB/c and C57BL/6 mice are driven by differences in neurological DEGs at each time point, Log_2_ scores for specific neurological signaling (*GABRA1* and *GABRG2*) and repair (*MAGEE1* and *LPAR1*) genes were compared between BALB/c and C57BL/6 mice (**Figure 7C**). *GABRA1* expression is comparable at both naïve and 14dpi timepoints and significantly increases compared to C57BL/6 mice at both 28 and 56dpi (**Figure 3C**). In contrast, neuronal signaling *GABRG2* expression is significantly elevated in BALB/c mice at all time points (**Figure 7C**). Neuronal signaling and repair genes *MAGEE1* and *LPAR1* also follow similar patterns of expression at each time point and are significantly elevated in BALB/c mice at each stage of infection (**Figure 7C**). Taken together, these results collectively demonstrate differences in timing and expression of genes, immune cell recruitment, and pathway activation in resistant BALB/c mice compared to B6 mice indicating decreased neuropathology and enhanced repair.

## Discussion

In this study, we utilized a targeted approach to analysis of gene expression and investigated >1500 genes associated with neurological and immunological processes by direct counting of RNA transcripts without the need for amplification *via* NanoString technology (Geiss et al., 2008). We determined the changes in these host genes over time in parallel with changes in *T. gondii* developmental-specific genes. While much is known about changes in gene expression at specific stages of infection *via* commonly used sequencing techniques like RNAseq, there is a gap in knowledge regarding how gene expression fluctuates as infection progresses through chronicity and how these changes relate to development of the parasite (Montoya and Liesenfeld, 2004; Jia et al., 2013; Ngô et al., 2017). In addition, we compared gene expression changes between susceptible and resistant mouse strains with specific focus on differences in neuropathology and neuroinflammatory signatures. Results demonstrate a dramatic shift in both previously demonstrated and novel gene expression relating to neuropathology and neuroinflammation as chronic infection progresses and reveals possible pathways of inflammation resolution.

The establishment and maintenance of chronic infection involves complex changes as infection progresses from parasite entry and formation of cysts early during infection to long-term control of encysted parasites *via* immune cell recruitment and cytokine production leading to subtle worsening of neuropathology as infection progresses through the mid- and late chronic stages (Liesenfeld et al., 1996; Denkers, 1999; Lee and Kasper, 2004; Lachenmaier et al., 2011; Feustel et al., 2012; Lilue et al., 2013; Hong et al., 2017; Radke et al., 2018; Graham et al., 2020; Mukhopadhyay et al., 2020). Our chosen timepoints represent each of these stages to capture as much directional change in gene expression as possible. Initial results demonstrate that despite the establishment of chronic infection seen by stabilization of Toxoplasma genes by 28dpi, there continue to be changes in overall gene expression relating to neuropathology, neuroinflammation, and inflammation at varying stages of chronicity. The multitude of signature changes suggests an initial activation of neuropathology and neuroinflammation phenotypes by 14dpi. This switch becomes more pronounced as chronicity progresses even while immune parameters between 4 and 8 weeks post infection steady. 454 DEGs remain shared between time points indicating conserved transcriptomic changes as chronic infection progresses, with fewer specific genes for each time point.

Previous analyses of parasite genes at different developmental stages have established some developmental specific gene signatures of the parasite as it transitions from disseminating tachyzoite to cyst-forming bradyzoite (Hong et al., 2017; Radke et al., 2018; Garfoot et al., 2019; Goerner et al., 2020; Krishnan et al., 2020; Waldman et al., 2020). Evaluating selected genes whose expression corresponds with either constitutive, tachyzoite, or immature/mature bradyzoite phenotypes using ToxoDB, we identified gene expression during chronic infection that supports the phenotype of maturing cysts and little reactivation to tachyzoite replication even in a susceptible mouse model. The genes were selected based on their percentile of expression in each growth stage of *T. gondii*, for example, *BAG1* is expressed at the 91^st^ and 100^th^ percentile in ME49 early and late-stage bradyzoites but only at the 15^th^ percentile in tachyzoites making it a bradyzoite associated gene. The variability between biological replicates is greater during analysis of Toxoplasma specific genes (**Supplementary Figure 2**). This likely reflects the non-homogeneous pattern of cyst location in the brain as well as technical limitations due to overabundance of host material compared to parasite. Our results show that the constitutively expressed tachyzoite genes *ROP18, GDA1/CD39*, and *ADF* remain lowly expressed throughout the course of chronic infection. The transitional gene *GRA12* is upregulated at 56dpi indicating possible continued cyst formation in line with a general low level increase in cyst numbers overtime (Sinai et al., 2016). Even though neuropathology scores worsen in the C57BL/6 strain, these data do not suggest that significant parasite reactivation is the cause. Most bradyzoite-specific genes such as *SRS35A, BAG1*, and *LDH2* accumulate over time and remain dominant as infection progresses with increased presence of late-stage bradyzoites and support a maturation of cysts in the brain (Watts et al., 2015; Garfoot et al., 2019; Goerner et al., 2020).

Our results confirm previous research demonstrating immune response and canonical T cell activation while also showing accumulation of macrophages and continued proliferation and activation of CNS microglia. While cell type profiling and pathway analyses used in this study identify the most significantly changed cell types and broad pathway changes, other cell types and more specific pathways can be excluded or overlooked due to low probe counts. In the future, additional genes relating to cell types and pathways of interest such as neurons and astrocytes could be added and analyzed. We confirm classical immune pathway activation that demonstrates possible stabilization over time. Recent work has demonstrated similar increases in these pathways between the acute and chronic stage of infection after infection with *T. gondii* oocysts following GO analysis of RNAseq (Hu et al., 2020). In addition, we provide new in-depth analysis of *IFNγ* expression and downstream IFNγ-signaling genes *STAT1, GBP2*, and *ZBP1* in the context of chronic infection in the brain. Specifically, the differences in timing of expression of these genes suggests stage-specific, IFNγ-dependent mechanisms of parasite control.

While our results offer further insight into the kinetics of expression for genes previously associated with chronic *T. gondii* infection, our analysis also reveals novel genes associated with neuropathology and neuroinflammation that have not been explored. Specifically, results demonstrate significant upregulation of the genes *C4A, CTSS, IFITM3* and *PSMB8* and downregulation of *ATF2* and *NRG3* across all stages of chronic infection. *C4A* works in conjunction with *C3A* and *C5A* to activate the complement cascade and together, these genes trigger the degranulation of mast cells and basophils and increase vascular permeability in the context of the inflammatory immune response. In addition, *C4A* has been associated with roles in schizophrenia (Liesmaa et al., 2018; Melbourne et al., 2018; Prasad et al., 2018; Ji et al., 2019). While complement activation has been previously demonstrated in the brain during Toxoplasma infection, the common functions of *C4A* point toward a possible role in increasing vascular permeability gradually during chronic infection for the continued infiltration of peripheral immune cells into the brain (Xiao et al., 2016; Huang et al., 2019). Interestingly, BALB/c mice experience significantly less expression of *C4A* at each infection time point compared to C57BL/6 mice which is an indication that there is decreased vascular permeability in these mice compared to B6. *CTSS* encodes for cathepsin S that is expressed by neurons and is required for elastase activity in alveolar macrophages (Ji et al., 2018; Doherty et al., 2019). It has also been demonstrated that *CTSS* participates in both the production of pro-inflammatory cytokines interleukin 6 (IL-6), interleukin 8 (IL-8), tumor necrosis factor-α (TNF-α), and interleukin-1β (IL-1β) during ocular inflammation (Klinngam et al., 2018) as well as the degradation of antigenic proteins to peptides during antigen presentation (Hughes et al., 2016; Klinngam et al., 2018). The pattern of *CTSS* expression at each stage of chronic infection suggests a potential role for this gene in pro-inflammatory cytokine production, neuronal function, and continued antigen presentation as part of the immune response to *T. gondii* infection. There are no known functions of *IFITM3* during *T. gondii* infection, but is important for anti-viral effector functions *via* inhibition of viral protein synthesis and shuttling incoming virus particles to lysosomes for degradation (Lee et al., 2018; Appourchaux et al., 2019; Bedford et al., 2019; Kenney et al., 2019; Spence et al., 2019). *IFTIM3* plays a role in viral infections through IFN signaling, indicating possible novel anti-parasitic functions of this gene during *T. gondii* infection. *PSMB8* is involved in the degradation of cytoplasmic antigen processing to generate MHC I binding proteins *via* stimulation by IFNγ production, provides instructions to make one subunit of immunoproteasomes responsible for helping in response to infections, and also regulates glioma cell migration proliferation and apoptosis (Agarwal et al., 2010; Basler et al., 2018a; Basler et al., 2018b; Yang et al., 2018). While previous work on PSMB8 has produced minimal data in relation to Toxoplasma infection (Agarwal et al., 2010), further research specifically relating to chronic infection could elaborate on anti-parasitic functions in the CNS. In addition to regulating TNFα production, *ATF2* is a cAMP- or activator protein-dependent transcription factor that regulates the transcription of various genes involved in anti-apoptosis, cell growth, and DNA damage response (Falvo et al., 2000; Tsytsykova and Goldfeld, 2002; Watson et al., 2017; Meijer et al., 2020). While the effect of *T. gondii* infection on the transcription of TNFα has been studied in the past, the specific role of *ATF2* in the context of chronic *T. gondii* infection in the CNS has not been elucidated, and this gene may play a vital role in regulating TNFα production specifically in the brain or by CNS-resident cells (Leng et al., 2009). *NRG3* is well-known for aiding in the development and survival of neurons and oligodendrocytes in the brain in addition to aiding in excitatory and inhibitory synapse formation and controlling glutamate release by neurons (Müller et al., 2018; Wang Y. N. et al., 2018). While it has not been explored previously in *T. gondii* infection, the significant decrease in *NRG3* may be partly responsible for some infection-induced neuropathology such as increases in extracellular glutamate concentration and excess neuronal firing.

Our results demonstrate a progressive worsening of neuropathology and increased attempted repair of this pathology *via* upregulated angiogenesis genes as infection becomes more chronic in a susceptible mouse model. While most of these directional changes are not as dramatic as those associated with the immune response (**Figure 3**), these changes support previous research demonstrating neuropathology caused by infection (Cabral et al., 2016; David et al., 2016; Ngô et al., 2017), and a small group of pathways such as Cytokines, Activated Microglia, and Angiogenesis experience drastic changes as infection progresses to the late chronic stage. It is important to note that this worsening neuropathology is unlikely to be caused by reactivation of cysts in the brain even at the late stage of infection as seen by the low expression of tachyzoite genes and instead suggests more indirect neurological consequences of infection. However, the limitations in sensitivity of measuring parasite genes in samples dominated by host material means that we cannot rule out low level cyst activation despite a consistent late-stage bradyzoite phenotype over the duration of infection. Analysis results of astrocytic activation marker *GFAP* and neuronal signaling gene *GABRA1* support this idea of progressive worsening neuropathology. While the initial increase in astrocytic *GFAP* is expected as part of the initial response to CNS infection, what isn’t expected is the significant increase in expression that takes place in the late stage of infection after chronicity has been well established. *GABRA1*, along with *GABRB3*, is vital for the formation of GABA_A_ receptors and plays a pivotal role in GABAergic signaling that protects neurons in the brain from over-signaling, which can often lead to diseases such as epilepsy (McKernan et al., 1991; Tozuka et al., 2005). The significant drop in *GABRA1* expression during the mid- and late chronic stage could be a major target for treatment of infection-induced neuropathology and chronic infection in the future. In addition to supporting previous findings, our data also suggests a continuous progression of neuropathology specifically in the areas of transmitter production, function, and response, areas that were previously lacking in the context of chronic infection. However, while our results indicate progressive neuropathology as seen *via* decreased overall neural connectivity, we also see increases in genes and pathways such as myelination that suggest enhanced attempts at repairing and resolving this neuropathology.

Neuroprotective and repair mechanisms in other non-infectious models of CNS injury include remyelination to increase conductivity between previously damaged neurons, synaptic pruning by microglia to limit over-signaling, regeneration of axons, and various others. Recent work by the Harris lab indicates a role for local release of the DAMP IL-33 either by oligodendrocytes or astrocytes that is then required for protection against Toxoplasma (Still et al., 2020). Such innate sensing mechanisms may be needed not just for parasite recognition but also as a trigger to activate neural repair during late infection. The process of myelination has been explored extensively in the context of other models of CNS disease and injury (Gonsette, 2010; Kapitein and Hoogenraad, 2015; Felten et al., 2016; Li et al., 2017; Pałasz et al., 2017; Saab and Nave, 2017; Wang F. et al., 2018; Avila et al., 2020). The myelination pathway in this analysis is driven by several genes that are also associated with immune modulation. Throughout all stages of infection in the susceptible C57BL/6 strain, the increase in the myelination pathway is driven by the genes *Hexb, Tgfb1*, and *Cxcr4*. *Hexb* encodes enzymes in lysosomes that break down toxic substances and act as recycling centers (Mahuran, 1999; Ogawa et al., 2018). *Tgfb1* has been shown to promote remyelination in the adult CNS in addition to its classical pro-inflammatory role (Hamaguchi et al., 2019; Mota et al., 2020). *Cxcr4* is a known regulator of remyelination in other models of infection and CNS inflammation (Carbajal et al., 2011; Tian et al., 2018; Beigi Boroujeni et al., 2020). The steady increase in myelination genes throughout the kinetics of chronic *T. gondii* infection demonstrates a possible role of myelination in maintaining neuronal signaling and function to prohibit worsening of neuropathology as well as the possibility of remyelination to heal pathology or reorganize neuronal circuits (David et al., 2016). Thus, further experiments need to be conducted to determine if there is an increase in myelin or attempts at remyelination during infection which are now warranted following this data.

In contrast, the use of resistant BALB/c mice in these experiments reveals enhanced repair and resolution during infection allowing for insights into the role of neurological repair mechanisms in the context of resistance to chronic Toxoplasma infection. BALB/c mice exhibit differences in pathway activation of neuropathology and neurological health and function. Specifically, BALB/c mice experience downregulation in neuropathological and inflammatory pathways and upregulation in neurological transmitter function and neuronal structure compared to C57BL/6. This indicates that BALB/c mice may have differences in specific neurological genes that aid in maintaining neurological signaling and repairing damage done by infection. The differentially expressed neuronal signaling genes *GABRA1* and *GABRG2* and the repair genes *MAGEE1* and *LPAR1* were analyzed in BALB/c mice to answer this question. *GABRA1* and *GABRG2* are both critical for the formation of GABA_A_ receptors that regulate neuronal signaling (McKernan et al., 1991; Tozuka et al., 2005; Li X. et al., 2020), and these receptors are significantly upregulated in BALB/c mice compared to C57BL/6 mice indicating possible enhanced GABAergic signaling in BALB/c mice brains enabling better control of neuropathology caused by infection. *MAGEE1*, also known as melanoma-associated antigen E1 (MAGE Family Member E1) or *DAMAGE*, is plays roles in neuronal signaling and functions in tissue growth and repair (Albrecht and Froehner, 2004). *LPAR1* induces downstream signaling cascades that are essential for normal brain development and function of the nervous system and functions as intrinsic axon growth modulators for neurons after injury (Fink et al., 2017; Plastira et al., 2019). Both of these genes are also significantly upregulated in BALB/c mice at all chronic time points suggesting enhanced neuronal signaling, DNA repair, and regulation of regrowth of neurons during infection.

It is known that host background is a factor in resistance to *T. gondii* infection. While increased resistance to infection in BALB/c mice has previously been linked to enhanced immune response, less is known regarding how neuropathology signatures differ between resistant BALB/c mice and the more susceptible C57BL/6 model over the course of chronic infection. Results of merged BALB/c vs. C57BL/6 analysis demonstrate differences in gene expression at each stage of chronic infection. BALB/c mice exhibit their largest change in gene expression at 14dpi which then plateaus by 56dpi while the opposite is true for C57BL/6 mice. This indicates that BALB/c mice may experience pathological changes in the brain early during infection which are then at least partially rescued as indicated by the plateauing of gene expression at the later chronic stages. While 350-420 neuropathology and neuroinflammation genes are conserved between the mouse species during infection, BALB/c mice exhibit more downregulated DEGs compared to C57BL/6 mice. This indicates a definitive difference in neuropathology signatures between BALB/c and C57BL/6 mice that indicates less neuropathology in the resistant BALB/c strain.

In conclusion, the results of absolute RNA counts of inflammatory and neuropathology genes support previously published whole genome RNAseq data sets while demonstrating novel gene expression relating to neuropathology and neuroinflammation as chronic Toxoplasma infection progresses. It reveals possible pathways of resolution through the use of a targeted transcriptomic approach in a susceptible and resistant mouse model of infection. The use of this technology presents an additional analysis tool in the *T. gondii* scientific field with the possibility for more strategic analysis in the future. These results also open a doorway to a multitude of potential gene candidates that can be explored to elucidate possible therapeutic targets and to further the vital understanding of the kinetics of chronic infection in the brain.

## Data Availability Statement

The data presented in the study are deposited in the Gene Expression Omnibus (GEO) repository (link: https://www.ncbi.nlm.nih.gov/geo/), accession numbers GSE166828 (reference SuperSeries), GSE166825, GSE166826, GSE166827 (SubSeries).

## Ethics Statement

The animal study was reviewed and approved by the Institutional Animal Care and Use Committee (IACUC) University of California Riverside.

## Author Contributions

KB, DW, and EW designed and conducted the experiments. KB, AB, CD, and EW analyzed the data, and KB and EW wrote the manuscript. All authors contributed to the article and approved the submitted version.

## Funding

The authors received generous support from the following sources: NIH grants RNS072298 and RAI124682 (EW). EW also received support from UCR Graduate Division. CD is employed by Nanostring.

## Conflict of Interest

CD is an employee of NanoString.

The remaining authors declare that the research was conducted in the absence of any commercial or financial relationships that could be construed as a potential conflict of interest.
