# Supplementary material for: Targeted Transcriptomic Analysis of C57BL/6 and BALB/c Mice During Progressive Chronic Toxoplasma gondii Infection Reveals Changes in Host and Parasite Gene Expression Relating to Neuropathology and Resolution
Source: Front Cell Infect Microbiol. 2021 Mar 18;11:645778. doi: 10.3389/fcimb.2021.645778 (PMC8012756; doi:10.3389/fcimb.2021.645778)
Supplement: Supplementary file 1 [file DataSheet_1.pdf]

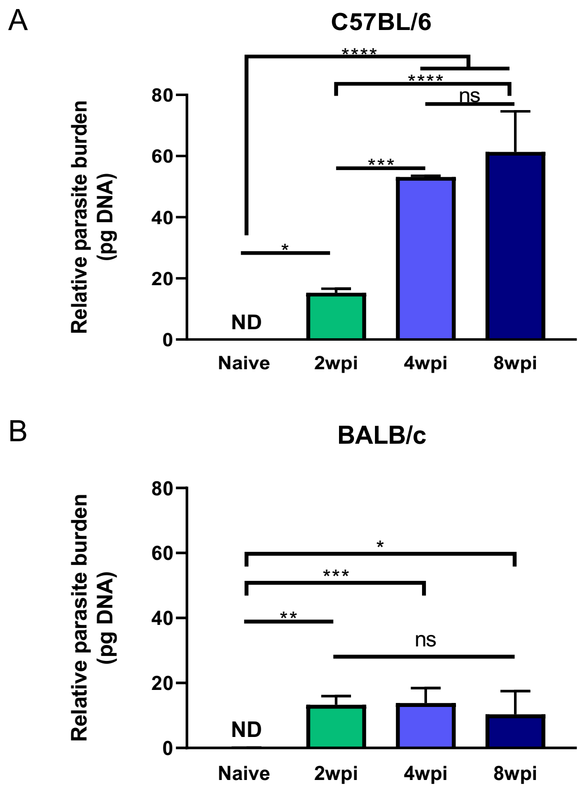

Supplemental Figure 1. Representative brain parasite burden for susceptible and resistant mouse strains. Quantified parasite burden from whole brain DNA via RT-PCR in susceptible C57BL/6 (n=4/time point) and resistant BALB/c (n=5/time point) mice. (**A**) C57BL/6 brain time point values were collected from multiple separate experiments and analyzed as an overall representation of parasite burden changes over chronic infection. (**B**) Brain samples from BALB/c mice collected for this study were tested and analyzed in parallel for parasite burden along with Nanostring nCounter analysis. Significance between timepoints determined by One-Way ANOVA using Multiple Comparisons (* = p-value <0.05, ** = p-value <0.01, *** = p-value < 0.001, **** = p-value < 0.0001).

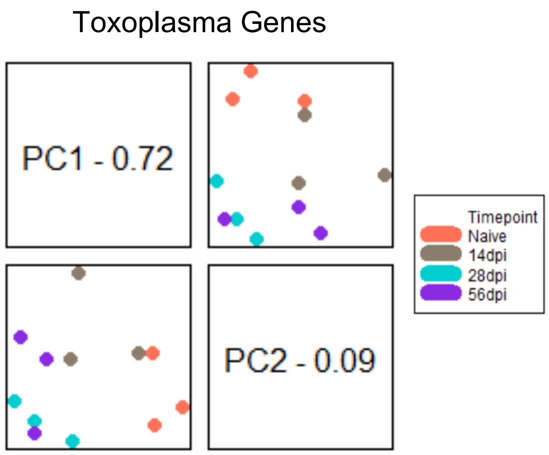

Supplemental Figure 2. Intragroup variation in relation to Toxoplasma genes. PCA plot of B6 biological replicates in relation to Toxoplasma gene expression at all time points. Numbers on axes represent percentage of variation in that component.
